# Prognostic Factors and Recurrence in Breast Cancer: Experience at the National Cancer Institute of Mexico

**DOI:** 10.5402/2012/825258

**Published:** 2012-07-05

**Authors:** A. Stankov, J. E. Bargallo-Rocha, A. Ñamendys-Silva Silvio, M. T. Ramirez, K. Stankova-Ninova, A. Meneses-Garcia

**Affiliations:** ^1^Breast Cancer Department, National Cancer Institute of Mexico, 14080 Tlalpan, DF, Mexico; ^2^National Cancer Institute of Mexico, 14080 Tlalpan, DF, Mexico; ^3^Department of Critical Care Medicine, National Cancer Institute of Mexico, 14080 Tlalpan, DF, Mexico

## Abstract

The purpose of this study was to analyze the prognostic and predictive factors that relate to locoregional or distant recurrences in breast cancer patients who have been treated at the National Cancer Institute of Mexico. Multivariate, time-dependent Cox regression analyses indicate that the pN status (positive versus negative lymph node; *P* = 0.003; HR (hazard ratio), 3.47; CI (confidence interval), 1.52–7.91) and the pathological complete response of the patient to neoadjuvant chemotherapy (yes versus no; *P* = 0.061; HR, 0.38; CI, 0.14–1.04) were important prognostic factors for recurrence.

## 1. Introduction

Breast cancer (BC) is the most common cancer among American women, with more than 200,000 new cases diagnosed each year. The lifetime risk for developing breast cancer has been reported to be as high as 12%, whereas the risk of death has been reported to be as high as 5% [1]. In Mexico in 2007, there were 16,340 reported hospitalizations for breast cancer and 4,872 breast cancer-related deaths. The introduction of mammography screening protocols in recent decades has facilitated the detection of breast cancer at increasingly earlier stages. For example, in the USA, stage 0 or I disease was found in 56% of cases in 1995 in comparison to only 45% in 1985 [2]. Today, we are able to detect BC at earlier stages; however, according to the literature, cases of locoregional and distant recurrences have been reported in 5 to 40% of cases. This wide range of reported results is probably due to inadequate axillary dissection, incomplete surgical technique, or suboptimal systemic treatment [3]. 

The involvement of the axillary lymph nodes (LNs) is the most important prognostic factor for recurrence in the early stages of BC according to the literature. Patients with cancer-positive LN have been reported to have a four to eight times higher mortality rate in comparison to patients with negative lymph nodes. There is also a direct correlation of positive LN status with the risk of distant recurrence [4].

In patients with negative LN, tumor size is an independent prognostic factor of breast recurrence. Patients with tumors that were smaller than 1 cm in diameter had an overall 5-year survival rate of 99%, whereas patients with tumors of 3–5 cm in diameter had a survival rate of 86% [5]. Tumor grade has also been widely accepted as a prognostic factor. The Scarff-Bloom Richardson (SBR) grading system takes into account the mitotic index and the differentiation and pleomorphism of the tumor. According to these characteristics, tumors can be well-, medium-, and poorly-differentiated (grades 1, 2, or 3, resp.). Grade 3 tumors have a relative recurrence risk of 4.4 times higher compared to the reference group. This prognostic factor has a significant relevance in patients with small tumors and negative LNs [6]. 

In clinical stage I, some studies have reported a recurrence risk of 38% when lymphovascular invasion (LVI) is present in comparison to 22% in cases where LVI is absent [7]; however, some studies have not found any differences [8]. Histologically, tubular, mucinous, tubulolobular, and cribriform breast tumors have the best prognoses. These tumors have a 10-year overall survival (OS) in 80% of cases [9]. Ductal, lobular solid, and mixed-type (ductal and lobular) tumors have a 10-year OS in only 50% of cases [10]. The worst prognosis occurs with inflammatory carcinoma, which has a 10-year OS in 30% of cases [11]. Hormone receptor status (HR) is both a prognostic and predictive factor in BC. A recent study has found that patients with positive HR had a higher percentage of 5-year DFS and OS [12]. Other authors have found that HR are a prognostic factor [13], and HR are a strong predictive factor in relation to adjuvant treatment with Tamoxifen or aromatase inhibitors [14, 15].

C-erb B-2 (Her2/neu) is present in 20–30% of BC patients [16]. Her2/neu is detected in tumors that are HR negative, have lymphatic infiltration, have high mitotic indices, and are BCL-2 negative. Patients with positive LNs and the presence or absence of Her2/neu expression have a 10-year OS in 50% and 65% of the cases, respectively.

Her2/neu expression can be used as a predictive factor of patient response to alkylating chemotherapy. Tumors with Her2 neu expression responded well and had a better survival rate in comparison to Her2 neu negative tumors, when treated with anthracycline-based chemotherapy [17]. Patients with Her2/neu expression poorly respond to Tamoxifen treatment [18].

In metastatic disease, the expression of Her2/neu is a predictor of patient response to Trastuzumab (Herceptin), wherein 4% of patients have a complete response, 17% a partial response, and 30% stable disease [19].

The grade of tumor proliferation can be measured in several different ways, and these different methodologies have been evaluated as prognostic factors. Some of these different methodologies include KI-67, the mitotic index, and the S-phase fraction [20–22]. Genetic profiling (Microarray) is typically used to identify gene expression profiles, which could help in the identification of prognostic or predictive factors in patients with small tumors and negative LN [23].

Another prognostic factor, albeit with less predictive power with is obesity. Studies have reported increased BC-related death rates among obese patients, in addition to a twofold increased risk of a contralateral breast cancer [24].

The intent of this study was to analyze the prognostic and predictive factors that are responsible for locoregional or distant recurrences in a group of patients treated at the National Cancer Institute of Mexico. 

## 2. Material and Methods

This retrospective transversal study included all of the patients who presented with a recurrence of a treated breast cancer from January 1 to December 31, 2007, at the National Cancer Institute of Mexico (INCan). A total of 11,144 patients were treated from January 1, 1986, to December 31, 2006, and a total of 60 of these patients showed a recurrence in 2007. The control group consisted of 60 other patients who were matched by clinical stage, time, type of treatment, and age. All of the information used in this study was obtained from the clinical archive of the INCan. The revised data used in this investigation consisted of pathology reports of true cut biopsies or the definitive operative material.

In the majority of cases, disease recurrence was diagnosed using imaging techniques (CT of the lung and abdomen using a Siemens Somatom Volumen Zoom 2003; nuclear bone scanning using a Siemens Gama Camera Series Signature e.cam; abdominal ultrasound using a Siemens Antares Acuson Premium; or X-ray of the chest wall using a Philips Super 80 Cp Diagnost 15). The rest of the cases were diagnosed by biopsies.

Both groups received adequate surgical interventions and neoadjuvant or adjuvant therapy during their primary treatments according to the guidelines of the NCCN. Pathology classification was conducted according to UICC/AJCC criteria. All tumors were histologically graded using a Scarff-Bloom-Richardson score. 

Vascular invasion was defined as tumor penetration into the lumen of an artery or vein. Vascular invasion should only be diagnosed when the tumor is demonstrated within one or both of a pair of vessels.

Extracapsular extension (ECE) was determined by a rupture of the axillary node membrane or when tumor cells were found in perinodular tissue. If the lymph node capsule was infiltrated but not penetrated, this was considered as ECE-absent.

The tissue expression of HR was determined by IHC. Immunohistochemical staining for the HER2 protein was performed using the DAKO HercepTest immunocyto-chemical assay (DAKO, Glostrup, Denmark) on 4-*μ*m-thick sections of formalin-fixed, paraffin-embedded material by the pathology unit of our institute and according to the manufacturer's instructions. This staining was scored as follows: 0 or 1+, no staining or membrane staining in <10% of tumor cells or barely perceptible and incomplete membrane staining in >10% of the tumor cells; 2+, weak to moderate complete membrane staining in >10% of tumor cells; 3+, intense and complete membrane staining in >10% of tumor cells. If IHC staining was indeterminate (2+), then the FISH method was conducted as a follow-up test. 

Recurrence was defined as an occurrence of disease six or more months after the last treatment.

## 3. Data Presentation and Statistical Analyses

Statistical analyses were performed using the Statistical Package for the Social Sciences software (version 15.0; SPSS, Chicago, IL, USA). The chi-squared or Fisher's exact test was used to compare categorical variables. Univariate and multivariate logistic regression were used to identify recurrence-associated factors. For categorical variables with multiple levels, the reference level was set to that with the lowest probability of the dependent variable. Variables with a *P* < 0.2 in the univariate analysis were entered into the model using a forward stepwise procedure. The results were summarized as odds-ratios (OR) and respective 95% confidence intervals (CI). Two-tailed *P* values < 0.05 were considered statistically significant. The area under the receiver operating characteristic curve (AUROCC) was used to evaluate the ability of the model to discriminate between patients in the recurrence and control groups [19]. A goodness-of-fit (Hosmer-Lemeshow) was calculated to assess the relevance of the logistic regression model [20]. The Kaplan-Meier method was used to measure the times of recurrence.

## 4. Results

In this series, the median age of the patients was 44.75 years (range 25–73) in the recurrence group (RG) and 44.3 years (range 25–74) in the control group (CG). The median tumor size was 5.08 cm (range 1–12 cm) in the RG and 4.35 cm (range 1–10 cm) in the CG. The median number of LN in the dissected material consisted of 16 LN in both groups (with a range from 2–35 in the RG and 2–38 in the CG). Patient characteristics are summarized in Table 1.

Some of the patients from both groups, depending on their clinical stage, were treated with chemotherapy, radiotherapy, and chemoradio therapy as a neoadjuvant treatment. In 42 patients (70%) of the RG, we used different regimens of neoadjuvant chemotherapy, depending on the patient and tumor characteristics. The aforementioned treatment regimens have been applied in recent years for the breast cancer. After treatment with neoadjuvant chemotherapy, the clinical responses of patients to these treatments were analyzed. Therein, 52% of patients in the RG, who presented with stable or progressive disease and were clinically or mammographically diagnosed following the neoadjuvant chemotherapy, were treated with chemoradio concomitant treatment or with radiotherapy alone. All of the patients were treated with surgery, and the type of surgery used in each case is presented in Table 2.

 The number of positive LNs and the tumor response therein in the group of patients who had been treated with neoadjuvant treatment were analyzed, and the results are summarized in Table 3.

All of the patients who had positive HR were treated with adjuvant hormone therapy for five years. Approximately 20 mg Tamoxifen was given to 43% of these patients (Table 4).

**Figure 1:**
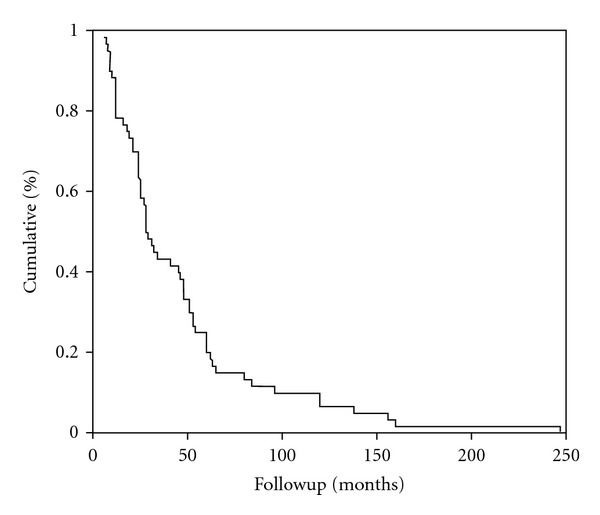
The median time of recurrence in our patients was 46.1 months (95% CI 34.8–57.4).

The median time of recurrence in our patients was 46.1 months (95% CI 34.8–57.4), as illustrated in Figure 1. In the RG, 23.8% of patients expressed Her2/neu.  Table 5 presents the recurrence prognostic values of the different tumor characteristics. 

The results of the univariate analyses to identify the independent risk factors of early recurrence in the breast cancer are presents in Table 6. 

On Table 7, the multivariate analyses were used to identify the independent risk factors of early recurrence in the breast cancer. 

## 5. Discussion

In this study, patients younger than 35 years of age (15% of the analyzed group) did not present with the usual tumor characteristics that have been previously reported such as big tumor size, positive LN, negative HR, and Her2/neu expression) [25]. This subgroup exhibited a tumor size, negative HR, positive axillary LN, and Her2/neu expression that were similar to those of the recurrence group (5.8 cm and 5.08 cm, 55.6% and 45.2%, 62.5% and 73.3%, and 25% and 23.8%, resp.). 

The clinical stage and skin involvement of patients in the analyzed group were used as criteria for comparisons to the CG, which is the reason why we did not include these attributes as prognostic factors. Nevertheless, 55% of group had locoregional advanced clinical stage tumor, whereas 42% had skin involvement at the time of diagnosis. 

In a study involving 70 patients with locally advanced clinical stage disease presentation (III-C) and who were treated with a combined modality treatment, a disease-free survival (DFS) and a median overall survival (OS) of 1.9 years and 3.5 years, respectively, were reported [26]. Statistical analyses demonstrated a longer DFS (2.8 years) in group of locally advanced clinical stage disease presentation (IIIA, IIIB, IIIC). This observation can be explained because of the aggressive locoregional treatment, which is standard treatment in our department for this group of patients. 

The group with advanced clinical stage disease presentation (IIB, IIIA, IIIB, and IIIC) were not statistically at higher risk for earlier recurrence in comparison to group with earlier stages of disease presentation (*P* = 0.084 IC 0.71–19.14), and this observation has been confirmed by a multivariate analysis. This result is not similar to a report from Montagna, who observed a 57% recurrence-free survival rate in the first two years of remission [27].

In this series, it was not possible to confirm the findings of Galea et al. [10], who observed that tumor histology predicted the recurrence rate or influenced the 10-year survival rate. 

It has been reported in the literature that tumor histological grade could be a relevant recurrence prediction factor, especially in small tumors with negative LN. In this study, we did not confirm the predictive value of histological grading, probably because the 85% of patients in RG and CG had large tumors with positive LN.

After performing a univariate analysis on the results, LVI was confirmed as a prognostic factor for tumor recurrence in this study, which agrees with the 60% higher mortality rate in patients when LVI is present, reported in the literature [28]; however, subsequent multivariate analyses exclude the role of LVI as prognostic factor for tumor recurrence. 

HR was observed not to be predictive of breast recurrence and disease-free interval when the two groups were compared after four years. This observation disagrees with the results reported by the NSABP B-06 study, which found that patients with positive HR had a higher percentage of 5-year DFS and OS, compared to patients with negative HR. Other authors have found HR to be a weak prognostic factor. This has been explained by the presumption that the predictive power of HR is only relevant in the first three years, because these tumors are usually well differentiated and their growth is slower [15]. When the positive HR was compared to the negative hormone receptors in the recurrence group, we found that 69% of patients with positive HR (estrogen, progesterone, or both) did not present with an early recurrence (up to 24 months from the termination of the initial therapy) (30.9 versus 69.1%  *P* = 0.014). Patients with negative HR did not show a statistically significant difference in terms of early or late recurrence. 

In this series, the histopathological reports could not distinguish gross from focal extracapsular extension (ECE), which is most likely why ECE was not identified as a prognostic factor. This finding agrees with those obtained by Gruber et al. [29].

Positive LN was identified as the most important prognostic factor for recurrence in this study. This observation agrees with those obtained by the NSABP study, which reported 5-year overall survival rates of 83%, 73%, 46%, and 28% for negative LN, 1–3 positive LNs, 4–12 positive LNs, and more than 13 positive LNs, respectively [4].

There were no differences in the expression of Her2/neu in the recurrence and control groups, and we could not confirm Her2/neu as a prognostic factor for recurrence in this study. These results are different from the findings reported in the literature [30, 31]. We believe that these results are because of the small number of patients with Her2/neu expression in both groups analyzed. 

Patients in the recurrence group exhibited a significantly lower complete pathological response in comparison to patients in the control group. We confirmed that patients with a complete pathological response had a lower risk of recurrence.

## 6. Conclusion 

In this study, the extent of nodal involvement and the complete pathological response to neoadjuvant chemotherapy were the most important predictors of breast cancer recurrence.

## Figures and Tables

**Table 1:** Baseline demographic and clinical characteristics.

	Recurrence group	control group
Age group		
>35	51 (85%)	51 (85%)
≤35	9 (15%)	9 (15%)
Clinical stage		
I	1 (1.67%)	1 (1.67%)
IIA	7 (11.67%)	9 (15.00%)
IIB	19 (31.67%)	22 (36.67%)
IIIA	15 (25.00%)	13 (21.67%)
IIIB	14 (23.33%)	15 (25.00%)
IIIC	2 (3.33%)	0
IV	1 (1.67%)	0
Unknown	1 (1.67%)	0
Skin involvement		
Yes	14 (23.33%)	14 (23.33%)
No	46 (76.67%)	46 (76.67%)
Clinical lymph node status		
N0	13 (21.67%)	13 (21.67%)
N1	33 (55.00%)	33 (55.00%)
N2	14 (23.33%)	14 (23.33%)
Clinical tumor size (cm)		
1	1 (1.67%)	3 (5.00%)
2	9 (15.00%)	4 (6.67%)
3	12 (20.00%)	16 (26.67%)
4	6 (10.00%)	11 (18.33%)
5	7 (11.67%)	9 (15.00%)
6	9 (15.00%)	9 (15.00%)
7	5 (8.33%)	3 (5.00%)
8	2 (3.33%)	2 (3.33%)
9	1 (1.67%)	1 (1.67%)
10	2 (3.33%)	1 (1.67%)
11	5 (8.33%)	0
Unknown	1 (1.67%)	1 (1.67%)
Histological type		
Ductal	51 (85%)	54 (90.00%)
Lobular	2 (3.33%)	3 (5.00%)
Ductal-lobular	6 (10.00%)	2 (3.33%)
Other	1 (1.67%)	1 (1.67%)
Histological grade		
Grade I	1 (1.67%)	2 (3.33%)
Grade II	21 (35.00%)	19 (31.67%)
Grade III	29 (48.33%)	27 (45.00%)
Unknown	9 (15.00%)	12 (20%)
Lymphovascular involvement		
Yes	26 (43.33%)	13 (21.67%)
No	34 (34%)	47 (78.33%)
Estrogen receptor status		
<20	29 (48.33%)	27 (45.00%)
20–49	8 (13.33%)	11 (18.33%)
50–100	10 (16.67%)	6 (10.00%)
>100	6 (10.00%)	8 (13.33%)
Unknown	7 (11.67%)	8 (13.33%)
Progesterone receptor status		
<5	26 (43.33%)	27 (45.00%)
5–30	5 (8.33%)	10 (16.67%)
31–50	2 (3.33%)	1 (1.67%)
51–100	12 (20%)	4 (6.67%)
>100	7 (11.67%)	9 (15.00%)
Unknown	8 (13.33%)	9 (15.00%)
Positive ER PR status	29 (54.7%)	31 (59.6%)
Negative ER PR status	24 (45.2%)	21 (40.3%)
Unknown	7	8
HER2/NEU expression		
Negative	32 (76.19%)	27 (81.81%)
Positive	10 (23.81%)	6 (19.19%)
Unknown	18	27
Extracapsular involvement		
No	52 (86.67%)	53 (88.33%)
Yes	8 (13.33%)	7 (11.67%)

**Table 2:** The type of surgery.

Type of surgery	Recurrence group	Control group
MRM Patey	39 (65.00%)	50 (83.33%)
MRM Madden	5 (8.33%)	3 (5.00%)
Segmentectomy	8 (13.33%)	6 (10.00%)
MR Halsted	6 (10.00%)	0
Total mastectomy + GS	2 (3.33%)	1 (1.67%)

Total	60 (100%)	60 (100%)

**Table 3:** The number of positive lymph nodes and the tumor responses in patients who were treated with neoadjuvant treatment.

Histopathology of axillary LNs	Recurrence group	Control group
Negative	16 (26.67%)	37 (61.67%)
Positive	44 (73.33%)	23 (38.33%)
Number of positive axillary LNs		
0	17	36
1	6	7
2	8	2
3	7	5
4	3	2
5	2	2
6	5	4
7	2	0
8	2	0
9	3	1
10	1	0
>10	1	0
>15	1	1
>20	2	0
Pathological response %		
Complete	8 (13.33%)	19 (31.67%)
90	15 (25.00%)	11 (18.33%)
80	2 (3.33%)	4 (6.67%)
70	3 (5.00%)	1 (1.67%)
60	2 (3.33%)	2 (3.33%)
50	5 (8.33%)	5 (8.33%)
30	0	2 (3.33%)
10	4 (6.67%)	3 (5.00%)
Unknown	21 (35.00%)	13 (21.67%)

Total	60	60

**Table 4:** Patients with positive hormone receptors who were treated with adjuvant hormone therapy for five years.

Hormonal treatment	Recurrence group	Control group
No	32 (53.33%)	27 (45.00%)
Tamoxifen	26 (43.33%)	27 (45.00%)
Aromatase Inhibitors	2 (3.33%)	6 (10.00%)

**Table 5:** The recurrence prognostic value of the tumor characteristics.

Tumor characteristics	Recurrence group	Control group	*P* value
Advanced clinical stage	52 (86.6%)	50 (83.3%)	0.609
Tumor size > 5 cm	36 (60%)	44 (73.3%)	0.121
Histological grade SBR 2; 3	50 (98%)	46 (95.8%)	0.610
Lymph vascular invasion	26 (43.3%)	13 (21.6%)	0.011
Positive estrogen receptor status	24 (40%)	25 (41.66%)	0.714
Positive progesterone receptor status	19 (31.66%)	24 (40.01%)	0.854
Positive ER PR status	29 (54.7%)	31 (59.6%)	0.572
Pathological lymph nodes status (positive versus negative)	44 (73.3%)	23 (38.3%)	<0.0001
Her2/neu expression	6 (19.19%)	10 (23.81%)	0.382
Pathological complete response	19 (31.67%)	8 (13.33%)	0.034

**Table 6:** Univariate analyses to identify independent risk factors of early recurrence in the breast cancer goodness of fit ((Hosmer-Lemeshow) *P* > 0.05 (×2), AUROOC = 0.77 (0.65–0.89) *P* < 0.001).

Variable	OR	IC	*P*-Value
Tumor size > 5 cm versus ≤ 5 cm	1.83	0.84–3.96	0.123
Positive ER PR status	0.80	0.38–1.69	0.572
Extracapsular involvement	1.16	0.39–3.44	0.783
Lymphovascular invasion	2.76	1.24–6.14	0.013
Pathological lymph nodes status (positive versus negative)	4.42	2.04–9.58	<0.0001
Pathological complete response	0.33	0.13–0.83	0.019

**Table 7:** Multivariate analyses to identify the independent risk factors of early recurrence in the breast cancer goodness of fit ((Hosmer-Lemeshow) *P* > 0.05 (×2), AUROOC = 0.72 (0.63–0.81) *P* < 0.001).

Variable	OR	IC	*P*-value
Tumor size > 5 cm versus ≤ 5 cm	1.80	0.77–4.20	0.169
Lymphovascular invasion	1.67	0.69–4.02	0.248
Pathological lymph nodes status (positive versus negative)	3.47	1.52–7.91	0.003
Complete pathological response	0.38	0.14–1.04	0.061

AUROCC: Area under the receiver operating characteristic curve.
